# Bile Flow Dynamics in Patients with Cholelithiasis: An Evaluation with Cine-Dynamic Magnetic Resonance Cholangiopancreatography Using a Spatially Selective Inversion-Recovery Pulse

**DOI:** 10.3390/tomography8020067

**Published:** 2022-03-16

**Authors:** Mayumi Higashi, Masahiro Tanabe, Kenichiro Ihara, Etsushi Iida, Matakazu Furukawa, Katsuyoshi Ito

**Affiliations:** Department of Radiology, Yamaguchi University Graduate School of Medicine, Ube 755-8505, Japan; m-tanabe@yamaguchi-u.ac.jp (M.T.); k.ihara@yamaguchi-u.ac.jp (K.I.); iida2283@yamaguchi-u.ac.jp (E.I.); matakazu@yamaguchi-u.ac.jp (M.F.); itokatsu@yamaguchi-u.ac.jp (K.I.)

**Keywords:** cine dynamic MRCP, bile, cholelithiasis

## Abstract

Background: A variety of pathophysiological changes in the biliary system occur in patients with cholelithiasis, but the changes in the bile flow dynamics in those patients remain unclear. The purpose of this study was to elucidate the changes in the bile flow dynamics in patients with cholelithiasis using cine-dynamic magnetic resonance cholangiopancreatography (MRCP) with a spatially selective inversion-recovery (IR) pulse. Methods: We retrospectively examined 25 patients with gallstones (gallstone group) and 69 patients without gallstones (non-gallstone group) who underwent abdominal MRI, including in- and opposed-phase T1-weighted images and cine-dynamic MRCP with a spatially selective IR pulse. The frequency and secretion grade of the antegrade and reverse flow of the bile on the cine dynamic MRCP images and the signal intensity ratio (SIR) of the gallbladder in the in- and opposed-phase T1-weighted images were evaluated. Results: The frequency and mean secretion grade of the antegrade bile flow were significantly higher in the gallstone group than in the non-gallstone group (*p* = 0.011 and *p* = 0.003), while no significant differences in those values of the reverse bile flow were found between the two groups. The SIR of the gallbladder in the T1-weighted gradient-echo in-phase images was significantly lower in the gallstone group than in the non-gallstone group (*p* = 0.004). Conclusions: Cine-dynamic MRCP with a spatially selective IR pulse can noninvasively visualize changes in the bile flow dynamics of patients with gallstones.

## 1. Introduction

Cholelithiasis is one of the most common diseases in the general population and affects approximately 10% to 20% of the population in the United States [1]. The causes of cholelithiasis are multifocal, including a complex interplay of sex-specific, genetic, lifestyle, and comorbidity-associated factors [2]. Although most people with cholelithiasis remain asymptomatic throughout their life, a minority of gallstones carriers can develop recurrent symptoms leading to cholecystectomy and various complications, such as choledocholithiasis, acute cholangitis, gallstone ileus, and acute gallstone pancreatitis [3]. The clinical course of cholelithiasis varies among individuals, and it is difficult to predict progression from asymptomatic to symptomatic disease using local factors (e.g., the number and size of the gallstones, alteration in gallbladder wall thickness, or gallbladder contractility) or general factors (e.g., the age, gender, or associated comorbidities) [4]. Therefore, the management of cholelithiasis should be individualized.

Several pathophysiological changes in the biliary system have been reported in patients with cholelithiasis. Even in the absence of any clinical and laboratory findings, gallbladder functions, such as filling and emptying, can be impaired in patients with cholelithiasis [5]. A recent study showed that the common bile duct pressure in patients with cholelithiasis was significantly higher than in those without cholelithiasis [6]. Previous studies have also shown that increased cystic duct resistance was associated with the pathogenesis of gallstones [7,8]. These changes in the physiological and pathological functions of the biliary system can affect the bile flow dynamics. Although the recognition of bile flow dynamics in patients with gallstones might play an important role in understanding the pathogenesis of cholelithiasis and determining appropriate patient management, the changes in the bile flow dynamics in patients with cholelithiasis remain unclear.

Cine-dynamic magnetic resonance cholangiopancreatography (MRCP) with a spatially selective inversion recovery (IR) pulse can directly and noninvasively visualize the physiological flow of bile [9,10]. Using this technique, some previous studies have evaluated the flow dynamics pattern of bile in a variety of pathological conditions, such as extrahepatic bile duct dilatation and post-cholecystectomy [9,11]. The present study, therefore, clarifies the changes in the bile flow dynamics in patients with cholelithiasis using cine-dynamic MRCP with a spatially selective IR pulse.

## 2. Materials and Methods

### 2.1. Study Population

This retrospective study was approved by our institutional review board, and the need for informed consent from the patients was waived. Between January and December 2019, 185 consecutive patients suspected of having pancreatic or hepatobiliary diseases based on their clinical history or having previously undergone ultrasonography or computed tomography (CT) underwent upper abdominal magnetic resonance imaging (MRI), including in- and opposed-phase T1-weighted images and cine-dynamic MRCP with a spatially selective IR pulse as the routine protocol for pancreatic and hepatobiliary MR examination in our hospital.

**Figure 1:**
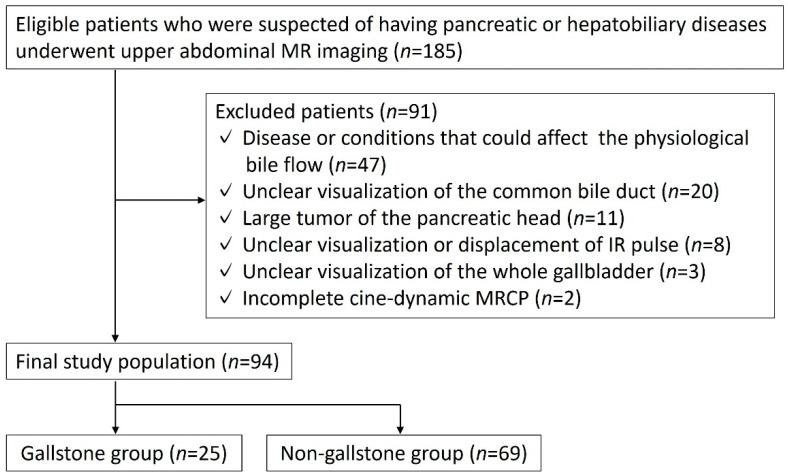
Flow chart of the included and excluded patients.

Of these patients, 91 were excluded due to the following reasons: disease or conditions of the biliary system or duodenal papilla that might affect the physiological bile flow other than cholelithiasis (*n* = 47) (post-cholecystectomy (*n* = 18), bile duct stone (*n* = 8), dilation of the common bile duct >10 mm due to unknown causes [12] (*n* = 8), primary sclerosing cholangitis (*n* = 3), benign biliary stricture (*n* = 2), tumor of the duodenal papilla (*n* = 2), primary biliary cholangitis (*n* = 1), IgG4-related sclerosing cholangitis (*n* = 1), cholangiocarcinoma (*n* = 1), postoperative state of pancreaticobiliary maljunction (*n* = 1), post-endoscopic sphincterotomy (*n* = 1) and postendoscopic papillectomy (*n* = 1)); unclear visualization of the common bile duct because of duct obstruction or artifacts (*n* = 20); large tumor or cyst of the head of the pancreas that interfered with the assessment of the bile flow on cine-dynamic MRCP (*n* = 11); unclear visualization or displacement of a spatially selective IR pulse (*n* = 8); unclear visualization of the whole gallbladder due to artifacts (*n* = 3), and incomplete cine-dynamic MRCP (*n* = 2). Ultimately, this study included 94 patients. Among these, we identified 25 patients (18 males, 7 females; median age, 72 (range, 45–84) years old) who had gallstones (gallstone group). The remaining 69 patients (32 males, 37 females; median age, 71 (range, 45–88) years old) were included as the non-gallstone group. The non-gallstone group included patients with pancreatic diseases (*n* = 48) (intraductal papillary mucinous neoplasms (*n* = 40), pancreatic neuroendocrine tumor (*n* = 3), chronic pancreatitis (*n* = 2), pancreatic gastrointestinal stromal tumor (*n* = 1), pancreatic lipoma (*n* = 1), and pancreatic cyst (*n* = 1)), with hepatobiliary diseases (*n* = 16) (adenomyomatosis (*n* = 6), hepatic hemangioma (*n* = 5), fatty liver (*n* = 3), and liver cyst (*n* = 2)) and no diseases (*n* = 5) (Figure 1).

### 2.2. MRI Technique

MRI was carried out using a 3.0-T MR system (Vantage Galan ZGO, Canon Medical Systems, Tochigi, Japan) with a 16-channel body coil. Patients were required to fast for at least 4 h before the MRI examination. At the beginning of MRI, 36 mg of manganese chloride tetrahydrate (a 250 mL package of Bothdel Oral Solution 10; Kyowa Hakko Kirin, Tokyo, Japan) was ingested to reduce the artifacts from bowel movement. First, a two-dimensional (2D), thick-slab MRCP image was obtained during a single breath-hold by using a fast advanced spin-echo sequence in the coronal plane, and this image was used as a reference image. The imaging parameters of this sequence were: slice thickness, 50 mm; repetition time (TR)/echo time (TE), 5000/507 msec; field of view (FOV), 35 × 35 cm; matrix, 512 × 512; echo train spacing, 6.5 msec; and bandwidth, 558 Hz/pixel. Then, using this MRCP image, a spatially selective IR pulse (inversion time = 2200 ms with width of 20 mm was set as perpendicularly as possible to the lower common bile duct to nullify the static bile signal [9]. With this imaging technique, the antegrade and reverse flow of the bile in the areas of a spatially selective IR pulse were observed as high signal intensity, while the static bile in these areas appeared dark. Conversely, the reverse bile flow outside the areas of a spatially selective IR pulse appeared as low signal intensity. MRCP with a spatially selective IR pulse was repeated every 15 s (scanning, 5 s; rest, 10 s) over 5 min (20 images total), and a series of these MRCP images was referred to as cine-dynamic MRCP with a spatially selective IR pulse.

The T1-weighted in- and opposed-phase images were obtained simultaneously with a 2D dual-echo gradient-echo sequence using the following parameters: repetition time/echo time (TR/TE), 125/2.52 msec (in-phase), 125/1.50 msec (opposed-phase); flip angle, 70°; slice thickness, 5.0 mm; acquisition matrix, 216 × 256; field of view (FOV), 360 mm; interslice gap, 1.4 mm; and band width, 976.5 Hz/pixel.

### 2.3. Image Evaluations

Three radiologists (M.H., M.T., and K.I., with 6, 19, and 33 years of clinical experience, respectively) reviewed cine-dynamic MRCP images independently. The radiologists were blinded to all patients’ clinical or laboratory data, and any discrepancies were resolved by consensus-based discussion. The MRCP images were evaluated for (a) the frequency of the antegrade and reverse bile flow observed during the 5 min MR imaging period (20 images) and (b) the secretion grade of the antegrade and reverse bile flow, which was defined according to the movement distance of the bile in the common bile duct using a 5-point secretion grade (grade 0 = no flow, grade 1 ≤ 5 mm, grade 2 = 5–10 mm, grade 3 = 11–15 mm, grade 4 ≥ 15 mm) [9]. The mean secretion grade of the bile flow was defined as follows: (total grade number)/20.

**Figure 2:**
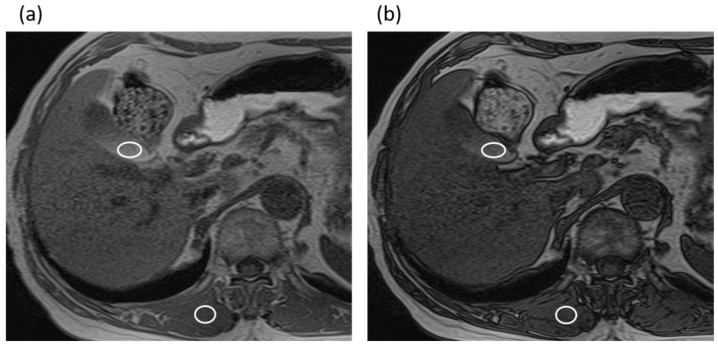
An 82-year-old man with intraductal papillary mucinous neoplasm in the non-gallstone group. (**a**) T1-weighted gradient-echo in-phase image. The gallbladder-to-muscle SIR was 2.12. (**b**) T1-weighted gradient-echo opposed-phase image. The gallbladder-to-muscle SIR was 1.72. The SRR of the gallbladder was 0.19.

The three radiologists measured the maximal diameter of the common bile duct perpendicularly to the long axis of the duct on MRCP images without the IR pulse by using an electronic caliper. The averaged common duct diameter of the two radiologists’ measurements was used for the data analysis. In addition, the three radiologists also measured the signal intensities (SIs) of the gallbladder (SI_gallbladder_) and paraspinal muscle (SI_muscle_) on the T1-weighted gradient-echo in-phase and opposed-phase images using operator-defined regions of interest (ROIs), in consensus. The signal intensity of gallbladder is often heterogeneous, forming gradational layering. Therefore, an effort was made to draw the ROI circles as large as possible in a high-signal area of the gallbladder on the in-phase images while avoiding the gallstones and artifacts, and the ROI was then copied and pasted on the opposed-phase images (Figure 2). The SI ratio (SIR) was then calculated from the SI_gallbladder_ and the SI_muscle_ as SI_gallbladder_/SI_muscle_ in each image. In addition, the signal reduction ratio (SRR) was calculated as follows: SRR = [(SIR in-phase) − (SIR opposed-phase)]/(SIR in-phase).

### 2.4. Statistical Analyses

All statistical analyses were conducted by using the SPSS software program (version 27.0 for Windows; IBM Corp., Armonk, NY, USA). Normality was tested by using the Shapiro–Wilk test. The Mann–Whitney *U* test was performed to compare the age and MRI measurements between the two groups. Spearman’s rank correlation coefficient analysis was used to assess the correlation between the MRI measurements. A multiple logistic regression analysis was conducted to examine the association of the presence or absence of gallstones with the MR measurements; analyzed MRI measurements included diameter of the common bile duct, SIR of the gallbladder on T1-weighted in-phase images, SIR of the gallbladder on T1-weighted opposed-phase images, frequency of the antegrade bile flow, and mean secretion grade of the antegrade bile flow. *p* Values of <0.05 were considered to indicate statistically significant differences. Interobserver agreement among the three radiologists was also evaluated by using weighted kappa values and interpreted as follows: 0.21–0.40, fair agreement; 0.41–0.60, moderate agreement; 0.61–0.80, substantial agreement; and 0.81–1.00, excellent agreement.

## 3. Results

The interobserver agreement among the three reviewers for the frequency of antegrade and reverse bile flow observed (reviewer 1 vs. reviewer 2, κ = 0.892; reviewer 1 vs. reviewer 3, κ = 0.886; reviewer 2 vs. reviewer 3, κ = 0.986) and the moving distance (secretion grade) of antegrade and reverse bile flow (reviewer 1 vs. reviewer 2, κ = 0.914; reviewer 1 vs. reviewer 3, κ = 0.907; reviewer 2 vs. reviewer 3, κ = 0.988) was excellent.

**Figure 3:**
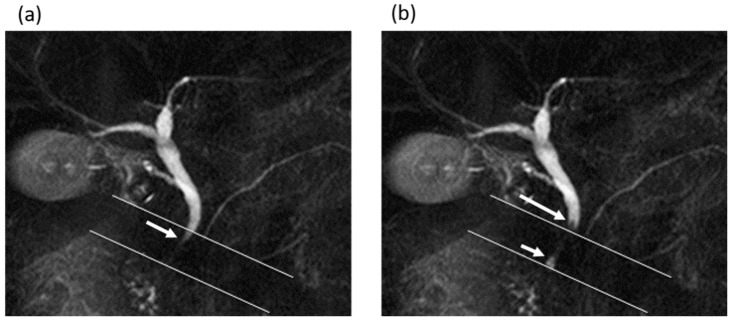
A 62-year-old man in the gallstone group. (**a**,**b**) Cine-dynamic MRCP images with a spatially selective IR pulse. (**a**) The antegrade bile flow appeared as high signal intensity (arrow) within the area of the IR pulse, showing a grading score of 2. (**b**) The reverse bile flow was seen as low signal intensity outside the area of the IR pulse (long arrow) and was also seen as high signal intensity coming from duodenal papilla side into the area of the IR pulse (short arrow). The grading score of reverse bile flow was classified as 1.

**Figure 4:**
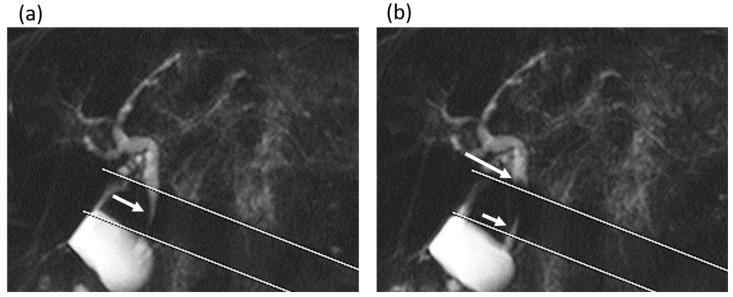
A 75-year-old man in the gallstone group. (**a**,**b**) Cine-dynamic MRCP images with a spatially selective IR pulse. (**a**) The antegrade bile flow was shown as high signal intensity (arrow) within the area of the IR pulse, showing a grading score of 4. (**b**) The reverse bile flow was seen as low signal intensity outside the area of the IR pulse (long arrow) and was also observed as high signal intensity coming from duodenal papilla side into the area of the IR pulse (short arrow). The grading score of reverse bile flow was categorized as 2.

In comparing MRI measurements between the gallstone group and the non-gallstone group (Table 1), the diameter of the common bile duct was significantly larger in the gallstone group (7 (range, 6–8) mm) than in the non-gallstone group (6 (range, 5–7.5) mm) (*p* = 0.015). The frequency and mean secretion grade of the antegrade bile flow were significantly higher in the gallstone group than in the non-gallstone group (frequency, 8 times (range, 4–11) vs. 3 times (range, 0–8); mean secretion grade, 0.55 (range, 0.25–0.85) vs. 0.2 (range, 0–0.4)), while no significant differences in those values of the reverse bile flow were found between the groups (frequency, *p* = 0.729; mean secretion grade, *p* = 0.703) (Figure 3 and Figure 4).

In analyzing the SIR of the gallbladder, the SIR on T1-weighted gradient-echo in-phase imaging was significantly lower in the gallstone group than in the non-gallstone group (1.2 (range, 0.77–1.51) vs. 1.62 (range, 1.2–2.12)), while there were no significant differences in the SIR for T1-weighted gradient-echo opposed-phase imaging between the groups (*p* = 0.210). In addition, the SRR of the gallbladder was significantly lower in the gallstone group than in the non-gallstone group (0.11 (range, −0.029–0.36) vs. 0.35 (range, 0.23–0.42)).

In the gallstone group, the frequency and mean secretion grade of the antegrade bile flow showed no significant correlations with the common bile duct diameter (frequency, *r* = −0.164, *p* = 0.433; mean secretion grade, *r* = −0.099, *p* = 0.638), the SIR of the gallbladder in T1-weighted in-phase imaging (frequency, *r* = 0.194, *p* = 0.353; mean secretion grade, *r* = 0.192, *p* = 0.358), or the SIR of the gallbladder in T1-weighted opposed-phase imaging (frequency, *r* = 0.209, *p* = 0.316; mean secretion grade, *r* = 0.135, *p* = 0.519) (Table 2).

In the non-gallstone group, the frequency and mean secretion grade of the antegrade bile flow also showed no significant correlations with the common bile duct diameter (frequency, *r* = 0.065, *p* = 0.593; mean secretion grade, *r* = 0.061, *p* = 0.619), the SIR of the gallbladder in T1-weighted in-phase imaging (frequency, *r* = −0.146, *p* = 0.232; mean secretion grade, *r* = −0.134, *p* = 0.273), or the SIR of the gallbladder in T1-weighted opposed-phase imaging (frequency, *r* = −0.097, *p* = 0.428; mean secretion grade, *r* = −0.092, *p* = 0.450) (Table 2).

A multiple logistic regression analysis showed that the presence of gallstones was only significantly associated with the mean secretion grade of the antegrade bile flow (*p* = 0.009, odds ratio = 3.972, 95% confidence interval = 1.409–11.198) and diameter of the common bile duct (*p* = 0.016, odds ratio = 1.424, 95% confidence interval = 1.067–1.899).

## 4. Discussion

Our study findings showed that the antegrade flow of the bile was greater in patients with gallstones than in those without gallstones. Furthermore, the diameter of the common bile duct was larger in patients with gallstones than in those without gallstones. The bile flow is likely regulated by the sphincter of Oddi, contraction of the gallbladder, and changes in intraductal pressure. Antegrade bile flow can be observed when bile flows out into the duodenum, triggered by the common bile duct pressure rising above the basal pressure of Oddi [13,14,15]. A recent study showed that the common bile duct pressure in patients with gallstones was significantly higher than in patients without gallstones [6]. As reported previously, gallstones can impair gallbladder filling, likely because of increased resistance to bile flow at the cystic duct [5,16]. Another study also indicated that the accumulation of bile in the gallbladder was delayed in association with sludge in the gallbladder and gallstones, suggesting an increase in internal pressure in the gallbladder and an eventual increase in bile volume in the common bile duct [17]. These data suggest that the increased observation of antegrade bile flow in patients with gallstones may be due to the increased pressure in the common bile duct, which is induced by impaired gallbladder filling and increased bile volume in the extrahepatic bile duct. A previous study showed that antegrade bile flow was more frequent in patients with cholecystectomy than in those without cholecystectomy [9,11], supporting our results.

In contrast, no significant differences in the frequency or mean secretion grade of the reverse bile flow were observed between patients with and without gallstones. A reversed bile flow is a physiologic phenomenon in the extrahepatic bile duct and may be a counteractive flow induced by contraction of the sphincter of Oddi. Our results suggest that the contractility of the sphincter of Oddi may even be preserved in patients with gallstones.

In the present study, the SIR of the gallbladder in T1-weighted gradient-echo in-phase imaging was significantly decreased in patients with gallstones than in those without gallstones. In T1-weighted imaging, the signal intensity of the bile varied greatly, depending on its concentration. In the fasting state, i.e., during an MRI, the absorption of water in the gallbladder causes an increased concentration of cholesterol and bile salts in the bile, leading to a reduced T1 relaxation time and increased T1 signal intensity of the bile [18]. Therefore, the decreased SIR of the gallbladder in patients with gallstones might be due to an insufficient bile concentration as a result of an impaired gallbladder function. In addition, the decreased SRR of the gallbladder in patients with gallstones may suggest a lower concentration of cholesterol in the bile than in patients without gallstones.

Our findings suggest that cine-dynamic MRCP with a spatially selective IR pulse may have potential utility for noninvasively evaluating changes in the bile flow dynamics in patients with gallstones. The management of cholelithiasis, including surgical intervention, depends on the presence of symptoms and complications [1,19]. However, symptoms, such as biliary pain, in patients with gallstones may not always be attributed to gallstones [20], so an objective assessment of the conditions of patients with gallstones may be needed to avoid unnecessary intervention and treatment. The assessment of the bile flow dynamics by cine-dynamic MRCP with a spatially selective IR pulse in combination with SIR measurements of the gallbladder may contribute to the estimation of the impaired gallbladder function, potentially proving useful for determining an appropriate management plan in patients with gallstones.

In the gallstone group, the frequency and mean secretion grade of the antegrade bile flow showed no significant correlation with the SIR of the gallbladder in T1-weighted imaging. This result suggests that the gallbladder’s ability to concentrate the bile in the gallbladder may not necessarily correlate with the increase in the antegrade bile flow due to impaired gallbladder filling in patients with gallstones. Alternatively, this result might be due to an insufficient fasting time. In the present study, the fasting time before MRI was established to be at least 4 h, which might not have allowed for a sufficient concentration of bile in patients with gallstones. A previous study suggested that gallbladder imaging should be performed after fasting for 8–12 h [18]. As a result, optimizing the fasting time before an MRI is necessary when assessing the MR findings of the gallbladder in a fasting state.

Several limitations associated with the present study warrant mention. First, since this study was retrospective and the number of patients in the gallstone group was relatively small, there may have been some selection bias. Second, we did not evaluate the association of the antegrade bile flow with the gallstone factors (such as the number, size, or composition of the gallstones) or patient factors (such as symptoms or associated comorbidities) in the gallstone group. Finally, we did not confirm the relationships between the increased antegrade bile flow in patients with gallstones and the pressure in the common bile duct and gallbladder pathologically. Further prospective clinical studies considering these factors with a large sample size of gallstones will thus be needed to validate our results.

## 5. Conclusions

Cine-dynamic MRCP with a spatially selective IR pulse can noninvasively visualize the changes in the bile flow dynamics in patients with gallstones. The antegrade bile flow was significantly increased in patients with gallstones.

## Figures and Tables

**Table 1:** Comparison of the MR measurements between the gallstone group and the non-gallstone group.

	Gallstone Group	Non-Gallstone Group	*p* Value
Number of patients	25	69	-
Age (year)	72 (62–77)	71 (65–78)	0.592
Common bile duct diameter (mm)	7 (6–8)	6 (5–7.5)	0.015
Frequency of observation of antegrade bile flow	8 (4–11)	3 (0–8)	0.011
Mean secretion grade of antegrade bile flow	0.55 (0.25–0.85)	0.2 (0–0.4)	0.003
Frequency of observation of reverse bile flow	3 (0–8)	3 (0–5)	0.729
Mean secretion grade of reverse bile flow	0.15 (0–0.5)	0.2 (0–0.35)	0.703
SIR in in-phase images	1.2 (0.77–1.51)	1.62 (1.2–2.12)	0.004
SIR in opposed-phase images	0.76 (0.65–1.2)	1.0 (0.76–1.42)	0.210
SRR	0.11 (–0.029–0.36)	0.35 (0.23–0.42)	0.004

Data are medians with the interquartile in parentheses. SIR = signal intensity ratio, SRR = signal reduction ratio.

**Table 2:** Correlation of the mean secretion grade and frequency of antegrade bile flow with the common bile duct diameter and with the SIR of the gallbladder in the gallstone group and non-gallstone group.

		Frequency of Antegrade Bile Flow		Mean Secretion Grade of Antegrade Bile Flow	
		*r*	*p*	*r*	*p*
Gallstone group	Diameter of common bile duct	−0.164	0.433	−0.099	0.638
	SIR in in-phase images	0.194	0.353	0.192	0.358
	SIR in opposed-phase images	0.209	0.316	0.135	0.519
Non-gallstone group	Diameter of common bile duct	0.065	0.593	0.061	0.619
	SIR in in-phase images	−0.146	0.232	−0.134	0.273
	SIR in opposed-phase images	−0.097	0.428	−0.092	0.450

SIR = signal intensity ratio.

## Data Availability

Not applicable.
